# Performance and Characterization of Bi-Metal Compound on Activated Carbon for Hydrogen Sulfide Removal in Biogas

**DOI:** 10.3390/molecules27249024

**Published:** 2022-12-17

**Authors:** Nurul Noramelya Zulkefli, Adam Mohd Izhan Noor Azam, Mohd Shahbudin Masdar, Nurul Akidah Baharuddin, Wan Nor Roslam Wan Isahak, Nabilah Mohd Sofian

**Affiliations:** 1 Department of Chemical & Process Engineering, Faculty of Engineering & Built Environment, UKM, Bangi 43600, Selangor, Malaysia; 2 Fuel Cell Institute, UKM, Bangi 43600, Selangor, Malaysia; 3 Research Centre for Sustainable Process Technology (CESPRO), Faculty of Engineering & Built Environment, UKM, Bangi 43600, Selangor, Malaysia

**Keywords:** bi-metal compound (BMC), removal hydrogen sulfide, hydrogen sulfide capture, adsorption, impregnated adsorbent

## Abstract

This study reports on the synthesis of bi-metal compound (BMC) adsorbents based on commercial coconut activated carbon (CAC), surface-modified with metal acetate (ZnAc_2_), metal oxide (ZnO), and the basic compounds potassium hydroxide (KOH) and sodium hydroxide (NaOH). The adsorbents were then characterized by scanning electron microscopy and elemental analysis, microporosity analysis through Brunauer–Emmett–Teller (BET) analysis, and thermal stability via thermogravimetric analysis. Adsorption–desorption test was conducted to determine the adsorption capacity of H_2_S via 1 L adsorber and 1000 ppm H_2_S balanced 49.95% for N_2_ and CO_2_. Characterization results revealed that the impregnated solution homogeneously covered the adsorbent surface, morphology, and properties. The adsorption test result reveals that the ZnAc_2_/ZnO/CAC_B had a higher H_2_S breakthrough adsorption capacity and performed at larger than 90% capability compared with a single modified adsorbent (ZnAc_2_/CAC). Therefore, the synthesized BMC adsorbents have a high H_2_S loading, and the abundance and low cost of CAC may lead to favorable adsorbents in H_2_S captured.

## 1. Introduction 

Hydrogen sulfide (H_2_S) is the most gaseous pollutant that is emitted through many industrial processes, such as petroleum refineries, food, industrial, paper and pulp manufacturing, and environmental facilities, such as landfill sites and wastewater treatment plants (where it is involved in the anaerobic digestion (AD) process) [1]. The AD process is an effective technology for the reduction in organic matter and the simultaneous production of energy, such as biogas. The presence of H_2_S may affect these industrial processes, human health, and environments. The foul odor of rotten eggs may also be fatal to humans if exposed at 320 ppm of H_2_S concentration.

Overcoming the removal of H_2_S in processing industrial and environmental facilities to endure in profitable technologies is challenging. Along with emitted H_2_S in biogas production, considerable differences in concentration are observed compared with processes of petroleum refineries [2,3]. The gaseous pollutant (H_2_S) is produced on the basis of different sources. The primary sources that trigger the production of H_2_S commonly involve bacterial activities in redox reactions of biochemicals, reactants used, and natural resources (i.e., waste sector, geothermal sources, marine sediments, agricultural waste, and coal seams) [1,4]. For example, Angelidaki et al. [5] showed that the emission of H_2_S for biogas production lies within the range of 1000–3000 ppm through manure digestion. However, the concentration of H_2_S in biogas production remains low compared with the natural gas industry with 10,000 ppm H_2_S production [1,3].

However, the AD process can be optimized using the following methods to improve the quality and quantity of biogas and digestate production: (i) substrate feeding strategy (simultaneous and appropriate feeding of various substrates), (ii) various waste pretreatments, (iii) suitable AD process conditions, and (iv) various additives [6,7,8,9,10,11]. Recent studies have demonstrated that incorporating the right additives to the bioreactor may be highly beneficial. In particular, the AD process stability can be increased along with syntrophic, metabolic, catalytic, enzymatic, and cation exchange biochemical activities as well as anaerobic biodegradability, biogas quality, and CH4 yields [9].

Several investigations have also revealed a significant possibility for improving the AD process and performance by adding other iron types [9,12]. Zhang et al. [13] indicate that iron concentrations as high as 5.65 gL^−1^ had no inhibitory effects on the AD process. The stability of the system and CH_4_ generation can be increased by using zero-valent iron additions in the right dosage and particle size, as demonstrated by Xu et al. [14]. Additionally, the inclusion of the iron chlorides FeCl_2_ and FeCl_3_ boosts the generation of CH_4_ and lowers H_2_S by precipitating the iron salt (FeS) [8,9,15].

The H_2_S production after the additives might be in certain concentration or even at low concentration; thus, other alternatives should still be considered to facilitate H2S removal. The tolerable concentration of H_2_S considering the process facilities/equipment and environmental and health issues indicates that the H_2_S exposure level must be below 10 ppm [16]. Moreover, certain devices, such as fuel cells, are recommended to have less than 1 ppm of H_2_S to prevent future catalytic poisoning.

Thus, several technologies, such as biotrickling filters [1,17], biofiltration [4], biological sulfur removal through microbe composition [18], mesoporous adsorption [19], and gas–liquid absorption have been introduced for the removal of H_2_S [20]. A commonly used well-known method is the Clauss process, which is applied in natural gas industries through a two-step reaction, oxidizing H_2_S to water and elemental sulfur. This method is commonly applied in high (>10,000 ppm) H_2_S.

Therefore, the adsorption technology through mesoporous materials is a recommended technique for the removal of H_2_S in the biogas system and has lower concentration compared with industrial fuel. However, using raw activated carbon (CAC) as an adsorbent, which frequently changes and leads to large amounts of secondary waste, must be considered. The capabilities of CAC in H_2_S adsorption were indelicate. Therefore, the development of high capability sorbents through metal oxide dispersion onto mesoporous materials can work even at a low temperature.

The removal of H_2_S mainly depends on selected mesoporous materials, including activated carbon normally used as a good carbon based on sulfur removal under moist condition [21]. Dhage et al. [22] stated that the presence of humidity did not affect H_2_S adsorption performance for silica-based materials. Thus, advanced studies on mesoporous materials, especially the activated carbon, show that the adsorption capability can be enhanced by surface modification (also known as impregnation process). That is, the high surface area and microporosity of activated carbon can undergo modification process with certain chemicals for the efficient adsorption of certain unwanted gases. Overall, the surface of adsorbents also promoted the adsorption–catalytic oxidation due to the interaction of surface chemistry. In addition to metal sulfides, this phenomenon also directed the supply of elements, such as S, SO_2_, sulfates, and sulfuric acid, in the system, through a minimal amount of oxygen and high humidity content [23,24,25,26].

Some studies used evolution adsorbents through metals and/or salts to convert H_2_S to metal sulfide also can enhance the H_2_S adsorption [27,28]. Additionally, basic compounds, such as KOH [23,29] and NaOH [29], were used to promote H_2_S oxidation. Adsorbents with metal oxides (Zn, Fe, and Cu), hydroxides, and carbonates were also extensively used in the current study of gas purification. The ligand molecules attached to the surfaces of adsorbents permit electrostatic or steric stability to stop adsorbents from aggregating. Investigating new ligands and functional groups is important to increase the stability, functionality, and adsorption capacity of adsorbents [6].

Thus, the chemicals used play a significant role in enhancing the capabilities of adsorbents, especially in captured H_2_S/CO_2_. However, the adsorption also depends on other critical criteria based on types of adsorbents, adsorption condition (initial concentration, breakthrough time, adsorption temperature and pH), adsorption characteristics (adsorption isotherm, kinetic model, adsorption thermodynamic, and adsorption mechanism), and adsorption capacity and their efficiency [6].

A previous study by Zulkefli et al. [19,30,31,32] showed that only certain chemicals are suitable for H_2_S elimination and the capabilities of adsorption might decrease with the involvement of other gases (i.e., CO_2_). Furthermore, a single impregnation still reveals a low adsorption capability toward H_2_S due to kinetic limitations factors [33,34,35,36,37,38]. Bi-metal compound (BMC) is one of the impregnation techniques that can be used for the enhancement of adsorbent capabilities rather than a single impregnation technique. Moreover, the modification on the carbon surface for BMC had different properties due to the single impregnation of adsorbents.

The adsorption performance may increase; thus, a study on the possible consequences could provide new information for the enhancement of H_2_S adsorption. Based on a study on fluoride removal through a dual impregnation method by Kalidindi et al. [39], researchers were motivated to expose a BMC to capture additional H_2_S gas. Moreover, a study by Lau et al. [40] proved that H_2_S adsorption capacity can be enhanced when the adsorbents are prepared (palm shell activated carbon, PSAC) with a mixture of cerium oxide (CeO_2_) and sodium hydroxide (NaOH). The breakthrough capacity for H_2_S reported 48.4 mg/g, which is 25 times larger than breakthrough capacity of raw PSAC. The combination of ZnO–CuO in mesoporous materials (silica and activated carbon) also shows significant synergic effect toward H_2_S adsorption capacity [21,22,36]. Additionally, the mixture of Zn–Cu–Al oxide for adsorbent preparation exhibits encouraging results considering H_2_S adsorption capability at low temperatures [41]. This condition also shows that Zn or Cu oxides on adsorbent surfaces can help adsorb the H_2_S even at low temperatures and prevent any reformate contaminants from entering the stream [42,43,44,45].

However, the study on synergic effect through a combination of metal acetate with metal oxide had not been deliberately investigated when these active phases are scattered onto activated carbon. Dispersing of dual metal oxides has been debated, but exploring the design of highly capable adsorbents is still important. Therefore, several adsorbents through BMC techniques were synthesized in this study by using ZnAc_2_ as the primary compound, which will be combined and mixed with basic compounds (i.e., KOH, NaOH) and metal oxides (such as ZnO). Hence, this BMC technique was synthesized and analyzed to discover the capability of certain chemical combinations toward H_2_S adsorption at ambient temperature. This study is also supported by different characterization analyses, including (i) scanning electron microscopic imaging, (ii) BET-specific surface, and (iii) thermogravimetric analysis (TGA) on BMC adsorbents samples, to establish the capability of H_2_S adsorption.

## 2. Results and Discussion

### 2.1. Surface Analysis of BMC Adsorbents

**Figure 1:**
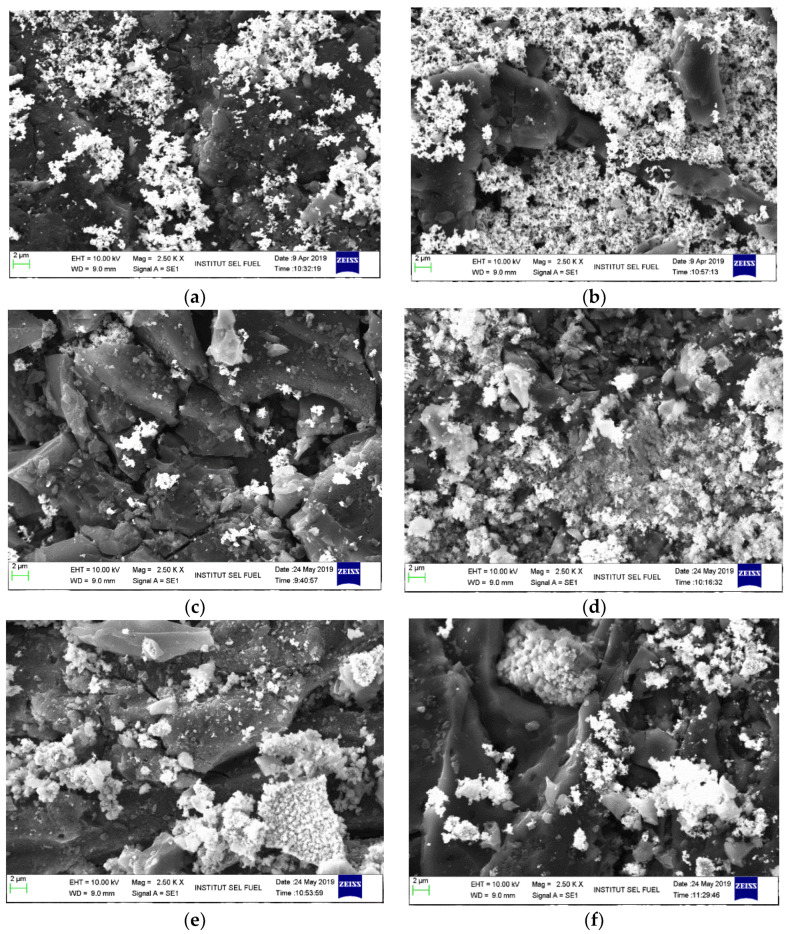
SEM micrograph images of a 2.5 kx (2 μm) adsorbent sample: (**a**) ZnAc_2_/ZnO/CAC_A (**b**) ZnAc_2_/ZnO/CAC_B (**c**) ZnAc_2_/KOH/CAC_A (**d**) ZnAc_2_/KOH/CAC_B (**e**) ZnAc_2_/ZnO/CAC_A (**f**) ZnAc_2_/NaOH/CAC_B.

Figure 1 shows the morphologies of fresh BMC adsorbents with a magnification of 2500× and a 2-micron scale. In addition to blurring and layering white spot images, the image reveals a dispersed agglomeration of anchoring BMC materials on the CAC surface, which formed crystal-like attachments on the surfaces of the adsorbents. However, the layering of BMC materials demonstrated considerable differences based on surface morphologies according to a comparative study in a previous investigation by Zulkefli et al. [19,30,31,32].

The study showed that the morphologies of raw CAC and single impregnation techniques exhibited clear and smooth surface images compared with BMC. The clear blurriness in this study was also expected due to the different ratios of chemicals used, which completely changed the morphology of the CAC surface. Hence, the morphology images from the modification on adsorbent surfaces confirmed that the element narrowed the originality of CAC porosity, which was predicted to improve the captured H_2_S.

Images of localized areas did not provide quantitative information on BMC content on adsorbent surfaces. Therefore, a study of elemental contents on the surface of BMC adsorbents through EDX analysis was conducted to obtain quantitative information. Table 1 shows the atomic weight % (wt.%) of the element contained in a specific area of fresh and exhausted adsorbents.

EDX analysis was performed on the elements C, Ca, Zn, O, K, and Na in all BMC adsorbents (Table 1). The table revealed that C content of ratio 1:1 BMC adsorbents was highest compared with a 2:1 ratio. The reduction in C contents was due to the high composition of the chemical amount covering the surface of adsorbents. The data confirmed that a high wt.% of Zn, O, K, or Na elements in a 2:2 ratio yielded a low wt.% of C elements. In addition, the presence of low Ca content in all BMC adsorbents confirmed that the main source of adsorbents was derived from activated carbon families [46].

The Zn content in fresh adsorbents shows an increment when a metal–metal composition (ZnAc_2_/ZnO) was used compared with a metal-based combination (ZnAc_2_/KOH or ZnAc_2_/NaOH). However, the Zn contents decreased after the adsorption process due to the Zn-S formation on the adsorbent surface. This formation also led to the continuous increase in S content after the adsorption process, as mentioned in exhausted adsorbents. However, with the formation of a strong S content bond, the S wt.% was still measured even after the purging process was introduced. The retention of the S atom after desorption also indicated a strong chemical bond between H_2_S, and the surface of the adsorbent will also affect the degradation issues in the study of adsorption–desorption cycles.

Similar results were obtained in a study by Isik–Gulsac et al. [46] due to the presence of S atoms on the surface of the adsorbents. Next, the O element for BMC adsorbents in KOH and NaOH was most utilized among other adsorbents. The presence of O elements may affect the positive adsorption performances, in which the highest O content in fresh adsorbents are probably the best adsorbents for H_2_S capture. This statement was confirmed in a previous study by Zulkefli et al. [19,30,31,32].

Surface area and pore structure are important factors involved in the performance of BMC adsorbents. The surface area was obtained by a measurement of the BET isotherm, while the pore volume and average pore volume were determined by the N_2_ adsorption isotherm at P/Po of 0.98. The pores contained all volumes of micropores, mesopores, and macropores. Upon modification of adsorbents, the BET surface area was decreased due to the blocking of pores by modified material, as previously observed in single modification adsorbents [19].

**Figure 2:**
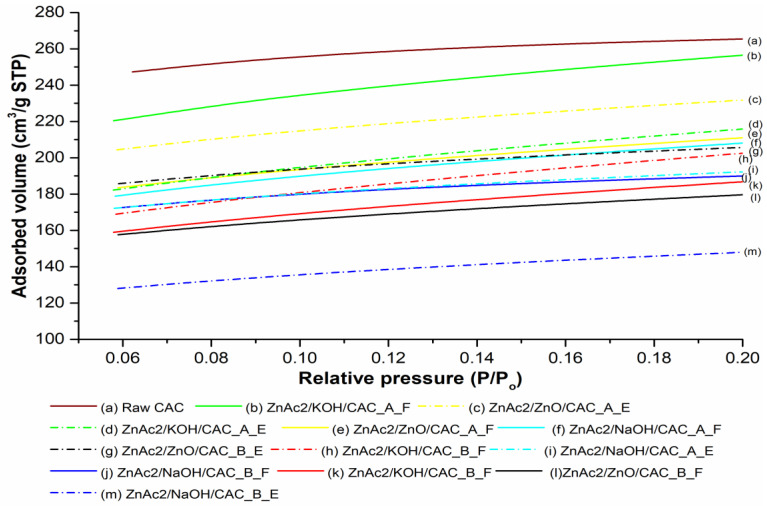
Nitrogen adsorption isotherms for modified porous adsorbents.

An analysis was conducted through N_2_ adsorption–desorption to determine the specific surface area and pore size distribution, and the analysis results are shown in Figure 2 and Table 2. The N_2_ adsorption isotherms of fresh and exhausted adsorbents are shown in Figure 2, which reveal that all the isotherms are Type 1(b) according to the updated classification of physisorption isotherms [40]. The Type I profile is concave to the relative pressure (p/p_0_) axis, which sharply rises at low relative pressures and reaches a plateau (also known as Langmuir isotherm). The Langmuir isotherm is acquired from the monomolecular adsorption of gas by porous solid materials [47]. Moreover, this Type 1(b) profile is typical of microporous materials with micropore diameter smaller than <2.5 nm [48,49].

The amount of N_2_ rapidly increases at the early stage of adsorption due to adsorption into micropores; hence, the slope of the adsorption curve is steep due to the profile shown in Figure 2, demonstrating an increase from 0 to 0.05 cm^3^/g at STP. Nevertheless, adsorption only occurred on the external surface of porous adsorbents after the adsorption into the micropores had been completed. Of all the fresh adsorbents, Zulkefli et al. [19] stated that the raw CAC adsorbed more N_2_ than modified porous adsorbents due to its large surface area, large pore volume, and micropore size.

Additionally, all modified adsorbents adsorbed less N_2_ because the deposited BMC materials (fresh adsorbents) and H_2_S components (exhausted adsorbents) partially filled or blocked the micropores of the adsorbents. The excess BMC material deposited on the surface of the adsorbents also resulted in low N_2_ adsorption, as confirmed by Ghazali et al. [50], who showed that the BMC materials blocked the mesopores, preventing the N_2_ from diffusing inside the pore.

Following the N_2_ adsorption experiments, the surface properties of the porous adsorbents (i.e., BET, average pore size, and pore volume) were determined as shown in Table 2. The BET surface area was considerably high and fell within reasonable CAC range (500–1500 m^2^/g). Commonly, with a low surface area of BET resulting in a high surface area of adsorbents, the capability of adsorbents to adsorb certain quantities of preferred gases may be enhanced. Several studies have confirmed that modified adsorbents capable of adsorbing high numbers of gases had low BET surface area and demonstrated the best performance in capturing gases [51,52]. Moreover, this reduction phenomenon of pore size and specific surface area, which was reported by Bai et al. [53], is related to the formation of mesopores from the combination of some micropores of activated carbon or the transformation of pore structure via coagulation and an excess of BMC materials.

The ratio number affected the BET surface area, wherein ratios of 2:1 provided a larger surface area compared with ratios of 1:1. Ratios 2:1 for ZnAc_2_/ZnO/CAC_B resulted in a better surface area (624 m^2^/g) compared with that of ZnAc_2_/KOH/CAC_B (649 m^2^/g) and ZnAc_2_/NaOH/CAC_B (659 m^2^/g), which was assumed to capture additional H_2_S gases. The activating agents referred to the BMC chemicals used on the surface of the adsorbent, which led to the development of additional new pores and the widening of existing pores. As in ZnAc_2_/ZnO/CAC_B (0.2 cm^3^/g), the metal loaded on the surface of adsorbents resulted in a decrease in micropore volumes compared with other BMC adsorbents. The amounts of BMC loaded onto CAC covered the external and internal surface areas, facilitating the interaction between adsorbents and adsorbate. This research found that the impregnation phase allowed chemical deposition in the most internal pores, thus blocking the fine microporosity [54,55,56].

However, the average pore size for all the BMC adsorbents increased by up to 45% compared with the adsorbents in a previous study by Zulkefli et al. [19]. This increase indicated that the pores in BMC adsorbents were clogged, while the BET results confirmed that the BMC particles occupied the micropores of adsorbents in all BMC adsorbents. Regarding the IUPAC classification of pore dimensions, BMC adsorbents were signified as mesopores due to their average pore size between 20 and 500 Å. The mesopores were supposed to improve the capture of molecular gases [29]. The adsorption of H_2_S was related not only to the BET surface area and pore volume but also to the reaction between the adsorbents (H_2_S and CO_2_).

Nevertheless, the exhausted adsorbents show addition in BET adsorption for certain adsorbents, such as ZnAc_2_/ZnO/CAC_A, ZnAc_2_/ZnO/CAC_B, and ZnAc_2_/KOH/CAC_B, which indicated a reduction in specific surface area. The increase in pore size in exhausted adsorbents also confirmed that the pores were clogged with S atoms as highlighted in the EDX analysis. The reduction in specific surface area, pore size, and pore volume predicted that the adsorbent was effective in enhancing H_2_S adsorption due to excellent adsorbent bonding on the adsorbent surface.

**Figure 3:**
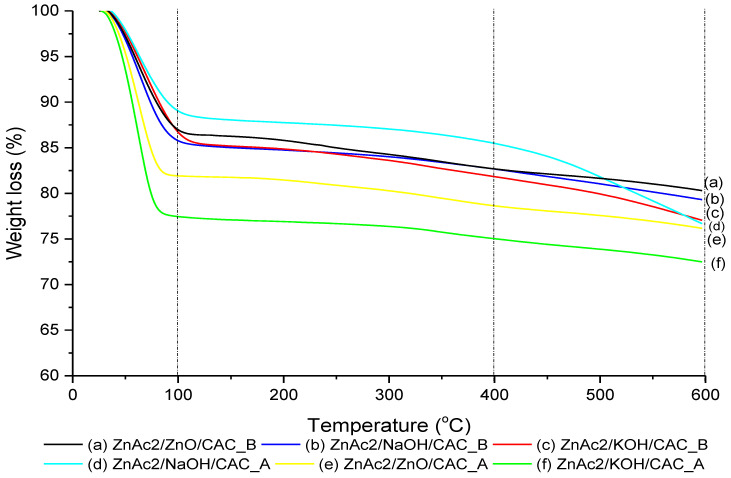
Thermogravimetric analysis weight loss curve.

TGA analysis was performed using various BMC adsorbents, as shown in Figure 3. The decomposition of adsorbents indicated by a considerable weight loss occurred in three main phases of temperature: (i) 25–100 °C, (ii) 100–400 °C, and (iii) 400–600 °C. The first temperature derivative around 100 °C was attributed to a weight loss of moisture contents of BMC adsorbents at approximately 11–23%. The decomposition of BMC materials observed at the second temperature derivative at approximately 400 °C revealed high thermal stability for all BMC adsorbents as indicated by the weight loss at 17% to 25%. However, the third derivative showed no major weight loss for all BMC adsorbents, except that for ZnAc_2_/NaOH/CAC_A, which had 23% weight loss primarily due to the decomposition of carbon in the composites.

The moisture contents in the TGA analysis show two different results: Less moisture contents for metal-based adsorbents were preferably used to adsorb H_2_S gas and highwater contents for basic compound enhanced the captured H_2_S. The ZnAc_2_/NaOH/CAC_A adsorbents then demonstrated high thermal stability up to 450 °C compared with other BMC adsorbents, which only depend at below 100 °C.

### 2.2. Performance of the H_2_S Adsorption-Desorption Test

**Figure 4:**
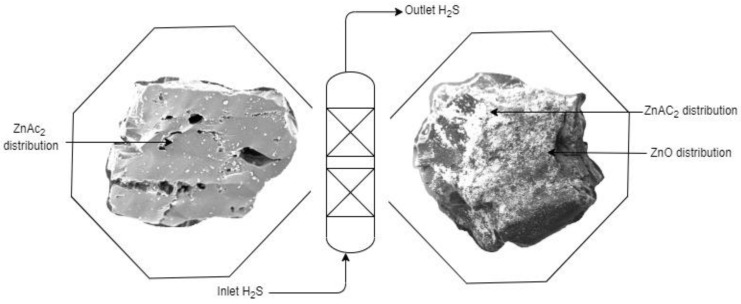
Differences in the distribution of chemicals on the surface of adsorbents.

A high number of chemicals used raises the chance of chemical distribution on the surface of the adsorbents, which was related to the high surface area. A high surface area increased the number of active sites and allowed additional H_2_S to bind to the sites, controlling the adsorption capacity. Figure 4 shows the difference in BMC adsorbents with a single impregnation of CAC through SEM images. The BMC adsorbents (ZnAc_2_/ZnO/CAC) demonstrated substantially blurry or white composition compared with ZnAc_2_/CAC, which suggests the extensive distribution of chemicals on the surface of adsorbents.

**Figure 5:**
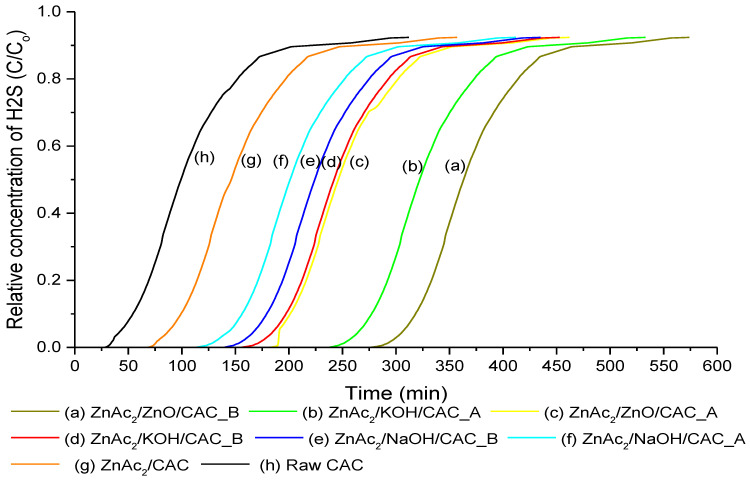
Breakthrough adsorption curve for different BMC adsorbents.

H_2_S adsorption–desorption was tested and evaluated using a single column adsorber unit with a constant flow gas feed (5.5 L/min) and an inlet concentration of commercial mixed gas. The adsorption was tested under dry conditions at 30 °C with gauge pressure at 1.5 bar. Then, the adsorption capacity for each of the BMC adsorbents was calculated based on Equation (1). Figure 5 and Table 3 present the adsorption capability of H_2_S exposure (adsorbate) on the BMC adsorbent surface from 0 ppm until the gas breakthrough from 1 ppm (relative concentration of H_2_S at 0.001) to up to 1000 ppm. The conceptual reaction of the H_2_S gas toward the adsorption system and the adsorbents included the following phenomena: (i) the adsorbate (H_2_S) was transferred from the tank onto the surface of the adsorbents and (ii) H_2_S was adsorbed on the adsorbent surface, in which the adsorbate reacted to the chemical on the activated carbon surfaces.

Among the BMC adsorbents, ZnAc_2_/ZnO/CAC_B (2.01 mg H_2_S/g) showed a high adsorption capacity followed by ZnAc_2_/KOH/CAC_A (1.73 mg H_2_S/g), ZnAc_2_/ZnO/CAC_A (1.33 mg H_2_S/g), ZnAc_2_/KOH/CAC_B (1.13 mg H_2_S/g), ZnAc_2_/NaOH/CAC_B (1.012 mg H_2_S/g), ZnAc_2_/NaOH/CAC_A (0.82 mg H_2_S/g), ZnAc_2_/CAC (0.37 mg H_2_S/g), and raw CAC (0.15 mg H_2_S/g). The raw CAC demonstrated the least adsorption capacity compared with single and BMC methods. Conversely, the BMC adsorbents were the most effective for capturing H_2_S and simultaneously competed with CO_2_ binds on the active site.

The BMC method significantly influenced the breakthrough time, the adsorption time, and the adsorption capacity compared with the single material modification and the raw CAC by up to 90%. The result also confirmed that the surface chemistry of adsorbents promoted the adsorption–catalytic oxidation mechanism for H_2_S. Furthermore, the performance of mixed Znac_2_/ZnO could be due to higher sulphidation/sulphation levels of metal acetate compared with metal oxide. Therefore, this result may be related to the performance of the metal oxide combination as indicated by Jiang et al. [41] and Balsamo et al. [57], which was tested with CuO-ZnO sorbents.

Therefore, the high number of chemicals on the surface of adsorbents showed good performance of H_2_S adsorption. The results reveal that the most effective adsorbent was ZnAc_2_/ZnO/CAC_B, which contained a high chemical load with a ratio of 2:1, potentially capturing additional H_2_S gas and competing with CO_2_ gas simultaneously. As mentioned in the early study by Zulkefli et al. [19], the CO_2_ contained in the feed systems determined a shift in the breakthrough profile toward shorter times compared to that without CO_2_ gas. However, ZnAc_2_/ZnO/CAC_B improved the adsorption capacity by up to 60% compared with the other BMC adsorbents.

The presence of CO_2_ in the systems inhibits a partial kinetic effect that is related to competitive CO_2_ and H_2_S gas adsorption onto active sites on the carbon surface [57]. Thus, increasing the number of chemicals improved the adsorption performance and enhanced the surface capability through the high surface area that was created. Conversely, O atom substitution and supported metal as mentioned in Table 1 also affected the chemical reaction on the surface of the adsorbents, which led to an increment in H_2_S adsorption capability.

### 2.3. Regeneration Adsorbent in H_2_S Adsorption–Desorption Test

**Figure 6:**
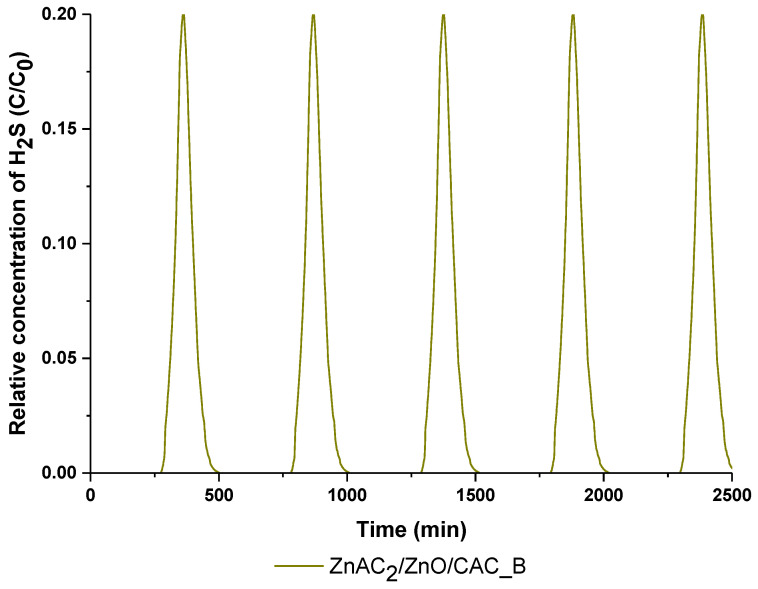
Regeneration profile of ZnAc_2_/ZnO/CAC_B.

Considering its highest H_2_S adsorption capacity, the ZnAc_2_/ZnO/CAC_B adsorbents were selected for further dynamic testing, which investigates the regeneration of the adsorbents. The regeneration of adsorbents is presented as in Figure 6 and Table 4. Across the entire investigation, the adsorption capacity slowly decreased based on the cycle numbers of adsorption–desorption that had been completed. Even after adsorption and desorption cycle, the adsorbent output at the first and third derivatives remained identical with fresh adsorbents. In any case, the presence of adsorbate on the surface of adsorbents during the adsorption–desorption process may have an effect on the adsorption capability of the next regeneration cycle [58]. A high temperature is used for eliminating the adsorbates (H_2_S) on the surface CAC. Hence, the formation of strong S-bond on the surface of adsorbents required a high temperature considering eliminating the adsorbates (H_2_S) [26].

Hence, the moisture content as demonstrated by TGA analysis showed two different results: Less moisture content in metal-based adsorbents was preferable to adsorb H_2_S gas and high water content of basic compounds enhanced the captured H_2_S. Competition between adsorbates (H_2_S and CO_2_) also affected the amount of captured H_2_S because basic compounds capture more CO_2_ gas than H_2_S, which influences the number of active sites available for H_2_S capture. The synergic contribution of carbonaceous support and chemical selection for the development of active phase can enhance the H_2_S adsorption capability [59]. Therefore, this study indicates a possible and potential applicability of metal–metal adsorbent from multicomponent stream for high H_2_S uptake, such as high adsorption efficiency of biogas.

## 3. Materials and Methods

### 3.1. Chemicals and Reagents

Granular type (3.0 to 4.2 mm) commercial CAC was supplied by Effigen Carbon Sdn. Bhd, Kapar, Malaysia. The chemicals used in this study were of analytical grade and were purchased through Friedemann Schmidt (Washington, USA). The selected BMCs were ZnO, ZnAc_2_, KOH, and NaOH. These materials were used as received, without further purification.

### 3.2. Preparation of Modified BMC/CAC

**Figure 7:**
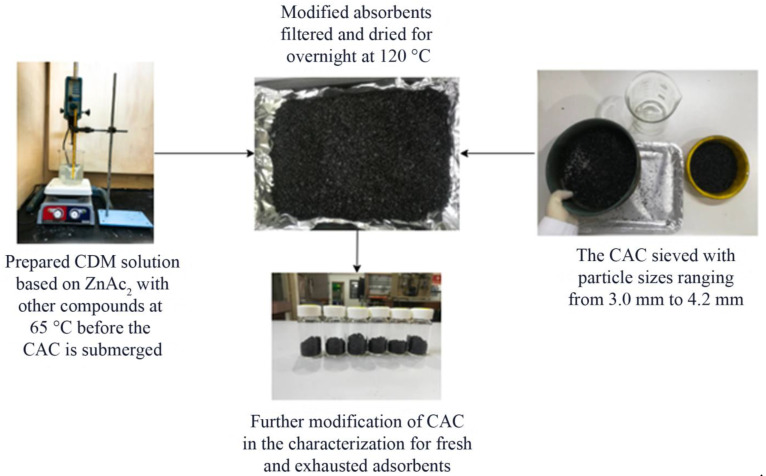
Adsorbent preparation steps.

Six sorbents were prepared and synthesized as in Figure 7 with chemical mixtures between chemicals Zn(CH₃CO₂)₂·2H₂O (or denoted as ZnAc_2_ throughout this study), zinc oxide (ZnO), potassium hydroxide (KOH), or sodium hydroxide (NaOH) by incipient wetness impregnation or in other terms (BMC technique). The 0.2 M of ZnAc_2_ was set as minimum prepared molarity and guideline for weight ratio preparation (1:1 and 2:1) toward ZnO, KOH, or NaOH. The molarity (0.2 M) was prepared through a study from Phooratsamee et al. [45] in 600 mL of distilled water. The study through investigation of ratio numbers for chemical mixture was conducted on the basis of a study by Balsamo et al. [57]. The 350 g of raw CAC was loaded into BC solution at 65 °C. The CAC was then soaked and constantly stirred for approximately 30 min to ensure that all the surfaces were fully covered with BMC solution. Next, the soaked CAC were filtered and washed several times with distilled water to eliminate any impurity before drying at 120 °C for overnight [51]. Thus, all the synthesized BMC adsorbents were labeled as in Table 5.

### 3.3. Characterization of Adsorbents

The fresh and exhausted BMC adsorbents were further analyzed through several characterization studies under a scanning electron microscope with energy-dispersive X-ray spectroscopy (SEM-EDX), specific surface area through BET, and TGA.

The surface morphology and chemical composition of BMC adsorbent particles were analyzed using a CARL ZEISS EVO MA10 and EDX scanning electron microscope (EDAX APOLLO X model), respectively. The analysis was conducted to visualize the details of adsorbent properties considering structural particles and atomic wt.% of elements present on the surfaces of adsorbents under an accelerating voltage of 10 kV. Further investigations on the properties of the adsorbents were conducted through the BET calculation method using Micrometric ASAP 2010 Version 4.0. The specific area and surface porosity were described through N_2_ adsorption–desorption isotherm. Next, the adsorbents were analyzed for thermal stability via TGA (TGA-50, manufactured by Shimadzu, Tokyo, Japan). The analysis observed weight loss over temperature changes, where 1 g of BMC adsorbents were heated at a heating rate of 10 °C/min under 20 mL/min of purified air flow up to 600 °C.

### 3.4. Real Test of H_2_S Adsorption–Desorption Process

Real testing of H_2_S adsorption was performed in a laboratory scale with a fixed-bed adsorber column with D_in_ = 0.06 m. A total of 155 g of BMC adsorbent was readily loaded into the adsorber column for testing with selected commercial mixed gas. A total of 1000 ppm H_2_S with 49.5 vol. % N_2_ and CO_2_ was fed into the column with constant flow rate (5.5 L/min) at T = 30 °C and gauge pressure set as 1 bar for each run. This adsorption method followed the previous study by Zulkefli et al. [19,30,31,32].

During the adsorption process, the H_2_S breakthrough gas concentration at the outlet stream was fixed at 1–10 ppm due to the tolerable range of the environment-exposed gas and fuel cell devices [60]. This outlet concentration was detected and recorded by using a portable H_2_S analyzer (model GC310), which was connected to the interface of the analyzer with the PC unit integrated into the monitoring software of the H_2_S analyzer.

Each of the adsorbents that undergo adsorption process were recommended to desorb the exhausted adsorbents with several steps involved in the desorption process. These methods normally practice for checking the capability adsorbents used in multiple cycles. Afterward, the desorption process for each of the adsorbents followed the setup preparation in a previous study by Zulkefli et al. [19,30,31,32], wherein the exhausted adsorbents undergo a purging process through three steps. First, the exhausted adsorbents were purged with a hot air blower for 30 min at 150 °C with a flow rate of 100 L/min. Second, the same operating parameters were applied into the column without temperature usage for 30 min. Third, the N_2_ gas was introduced into the column with 5.5 L/min for 30 min to purge out and stabilize the surface adsorbents before their use for the next adsorption–desorption process.

### 3.5. Calculation of Adsorption Capacity

A calculation was conducted on the basis of the computation presented in Equation (1) to measure the adsorption capacity of H_2_S, Q (mg H_2_S/g) [19,61]. The adsorption capacity was calculated on the basis of breakthrough time of H_2_S at the outlet stream in min ($\;T_{B}$), the fed flow rate in L/min (q), the mass adsorbent loaded into column in kg ($m_{ads}$), H_2_S feed gas concentration in kg/L, (C), molar volume at STP ($V_{M}$) which 22.4 L and molecular weight of H_2_S in kg/kmol ($MW_{H2S}$):

(1) $$\;Q=\frac{q\times T_{B}\times C\times MW_{H2S}}{V_{M}\times m_{ads}}$$

As in the adsorption–desorption process, a calculation based on degradation of adsorption capacity for each cycle was then conducted for the performance of adsorbents. This degradation study is also known as regeneration process for each cycle of adsorbents. Thus, the capability of selected adsorbents can be observed. Hence, a calculation considering percentage can compute differences in adsorption capacity in a previous cycle, $\;Q_{n}$, and current cycle, $\;Q_{n-1}$, through the following Equation (2) [19].

(2) $$\;Degradation=\frac{\;Q_{n}-\;Q_{n-1}}{\;Q_{n}}\times 100$$

## 4. Conclusions

Surface modification through certain amounts and combinations of chemicals can improve the surface properties of CAC. Further modification of adsorbent with BMC strongly promotes H_2_S chemisorption and improves the efficiency of H_2_S adsorption by adsorption capability based on breakthrough time. The presence of metal atoms also promotes the capability of adsorbents to adsorb more H_2_S gas compared with basic compounds with the presence of high O atoms, which preferably adsorb CO_2_ gas. In addition, the presence of S atoms in exhausted adsorbents confirms that some of the adsorbents are affected by H_2_S adsorption capacity due to chemical bonding between H_2_S and modified adsorbents. The high specific surface area of ZnAc_2_/ZnO/CAC_B enhanced the H_2_S adsorption performance with low moisture content due to the analysis in low weight loss percentage. Nevertheless, metal-based BMC adsorbents can be used to mitigate the competition between H_2_S and CO_2_ adsorption by enhancing the H_2_S adsorption capacity.

## Figures and Tables

**Table 1:** Contents of elements in fresh and exhausted adsorbents.

Adsorbents		C	Ca	Zn	O	K	Na	S
ZnAc_2_/ZnO/CAC_A	Fresh	56.1	0.3	29.1	24.5	0.0	0.0	0.0
	Exhausted	68.3	0.3	11.2	17.0	0.0	0.0	3.23
ZnAc_2_/ZnO/CAC_B	Fresh	28.1	0.4	45.5	26.1	0.00	0.0	0.0
	Exhausted	64.3	0.3	10.9	21.8	0.0	0.0	2.7
ZnAc_2_/KOH/CAC_A	Fresh	45.3	0.3	22.7	30.7	1.0	0.0	0.0
	Exhausted	49.8	1.8	11.1	26.9	7.3	0.0	3.2
ZnAc_2_/KOH/CAC_B	Fresh	29.0	0.5	36.6	31.0	2.9	0.0	0.0
	Exhausted	53.2	0.8	19.6	21.0	3.8	0.0	0.8
ZnAc_2_/NaOH/CAC_A	Fresh	35.4	0.6	11.6	40.2	0.0	12.1	0.0
	Exhausted	63.8	0.5	5.2	27.2	0.0	2.0	1.3
ZnAc_2_/NaOH/CAC_B	Fresh	10.4	0.5	27.7	44.5	0.0	17.0	0.0
	Exhausted	42.4	0.3	15.6	38.8	0.0	2.7	0.3

**Table 2:** Porous properties of fresh and exhausted adsorbents.

Adsorbents		BET Surface Area (m^2^/g)	Average Pore Size (Å)	Pore Volume (cm^3^/g)
ZnAc_2_/ZnO/CAC_A	Fresh	732	26.6	0.3
	Exhausted	797	25.4	0.5
ZnAc_2_/ZnO/CAC_B	Fresh	624	25.6	0.2
	Exhausted	702	23.0	0.4
ZnAc_2_/KOH/CAC_A	Fresh	890	26.5	0.3
	Exhausted	753	28.4	0.5
ZnAc_2_/KOH/CAC_B	Fresh	649	27.7	0.3
	Exhausted	710	30.0	0.5
ZnAc_2_/NaOH/CAC_A	Fresh	722	26.7	0.3
	Exhausted	657	23.8	0.4
ZnAc_2_/NaOH/CAC_B	Fresh	659	21.8	0.1
	Exhausted	512	26.0	0.3

**Table 3:** Adsorption capacity of H_2_S.

Adsorbents	Breakthrough Time, T_b_ (min)	Adsorption Capacity, Q (mg H_2_S/g)
ZnAc_2_/ZnO/CAC_A	182	1.33
ZnAc_2_/ZnO/CAC_B	276	2.01
ZnAc_2_/KOH/CAC_A	237	1.73
ZnAc_2_/KOH/CAC_B	155	1.13
ZnAc_2_/NaOH/CAC_A	113	0.82
ZnAc_2_/NaOH/CAC_B	139	1.01
ZnAc_2_/CAC	68	0.37
Raw CAC	28	0.15

**Table 4:** Degradation of adsorption–desorption cycle.

Number of Cycles	Breakthrough Time, Tb (min)	Adsorption Capacity, Q (mg H_2_S/g)	Degradation, %
1	276	1.49	0
2	276	1.49	0
3	276	1.49	0
4	274	1.48	0.67
5	273	1.47	1.34

**Table 5:** Description of prepared adsorbents.

Adsorbents	Ratio
ZnAc_2_/ZnO/CAC_A	1:1
ZnAc_2_/ZnO/CAC_B	2:1
ZnAc_2_/KOH/CAC_A	1:1
ZnAc_2_/KOH/CAC_B	2:1
ZnAc_2_/NaOH/CAC_A	1:1
ZnAc_2_/NaOH/CAC_B	2:1

## Data Availability

All relevant data are contained in the present manuscript.
